# Commensal Bacteria Regulate Thymic Aire Expression

**DOI:** 10.1371/journal.pone.0105904

**Published:** 2014-08-26

**Authors:** Akihito Nakajima, Naoko Negishi, Hiromichi Tsurui, Naomi Kadowaki-Ohtsuji, Keiko Maeda, Masanobu Nanno, Yoshitaka Yamaguchi, Nobuyoshi Shimizu, Hideo Yagita, Ko Okumura, Sonoko Habu

**Affiliations:** 1 Department of Immunology, Juntendo University School of Medicine, Bunkyo-ku, Tokyo, Japan; 2 Department of Pathology, Juntendo University School of Medicine, Bunkyo-ku, Tokyo, Japan; 3 Atopy (Allergy) Research Center, Juntendo University School of Medicine, Bunkyo-ku, Tokyo, Japan; 4 Yakult Central Institute for Microbiological Research, Kunitachi, Tokyo, Japan; 5 Advanced Research Center for Genome Super Power, Keio University, Tsukuba, Ibaraki, Japan; University of Tokyo, Japan

## Abstract

Commensal bacteria in gastrointestinal tracts are reported to function as an environmental factor to regulate intestinal inflammation and immune responses. However, it remains largely unknown whether such bacterial function exerts any effect on other immune organs distant from the intestine. In this study, the influence of commensal bacteria in the thymus, where T cell lineages develop into mature type to form proper repertoires, was investigated using germ-free (GF) mice and Nod1-deficient mice lacking an intracellular recognition receptor for certain bacterial components, in which a commensal bacterial effect is predicted to be less. In both mice, there was no significant difference in the numbers and subset ratios of thymocytes. Interestingly, however, autoimmune regulator (Aire) expression in thymic epithelial cells (TECs), main components of the thymic microenvironment, was decreased in comparison to specific pathogen-free (SPF) mice and Nod1 wild-type (WT) mice, respectively. In vitro analysis using a fetal thymus organ culture (FTOC) system showed that Aire expression in TECs was increased in the presence of a bacterial component or a bacterial product. These results suggest that through their products, commensal bacteria have the potential to have some effect on epithelial cells of the thymus in tissues distant from the intestine where they are originally harbored.

## Introduction

In the mammalian gastrointestinal tract, hundreds of species and large numbers of gut microbiota (commensal bacteria) are harbored within the lumen, and many of their functions have been reported, providing benefits including metabolic, homeostatic, and immune response in local areas. Of the intestinal immune response, the involvement of commensal bacteria has been well studied, and it is reported to affect the development of various immune cells such as Th17 and regulatory T (Treg) cells, NK cells, IgA-producing cells and dendritic cells in mice [1], [2]. Among these immune cells, commensal bacterial effects on Treg cells have been intensively studied as modulators of intestinal immune responses by regulating the nflammatory and/or allergic status in the intestine [1], [3]. In fact, Treg cells were reported to decrease in the intestinal mucosa when the mice are bred in germ-free (GF) condition lacking commensal bacteria [4], [5]. In more advanced studies, there is increasing evidence to show that commensal bacteria are executed as a modulator of innate and adaptive immune functions through their products and components [3], [6].

In those past studies, most were focused on the important role of commensal bacteria within the limits of the local intestine, but a few studies have indicated that commensal bacteria, and particularly their products, substantially affect the activation and/or development of immune tissues distant from the intestine.

Recently, bacterial components including peptidoglycan (PGN), a cell wall component found mainly in Gram-negative bacteria, have been reported to be circulating in serum [4], [7]. The Gram-negative bacterium belongs to a major member of commensal bacteria. Thus, we could predict that bacterial components can migrate to lymphoid tissues distant from the intestine including the thymus, where T cells develop and are selected positively and negatively in the context of MHC restriction. To determine the effect of commensal bacteria on the total immune system in the present study, we examined the thymus of GF and Nod1-deficient mice; the former lacks commensal bacteria, and the latter lacks a recognition sensor for certain bacterial components including PGN. As a target tissue, the thymus was selected because in general, it receives almost no immigration of developed and/or activated T cells already exposed to commensal bacteria in the intestine.

By comparing thymi of GF vs specific pathogen-free (SPF) mice, and of Nod1 -deficient vs Nod1 WT mice, thymocytes in cell number and subset proportions showed no significant difference. Interestingly, however, Aire expression in thymic epithelial cells (TECs) was decreased in both GF and Nod1-deficient mice. In vitro thymic lobe culture showed that bacterial components substantially enhanced Aire expression in TECs. Since Aire expression is considered to be important for the regulation of self-reactive T cells against tissue-specific antigens (TSAs), we will discuss the fate of self-reactive thymocytes in relation to Aire expression.

## Materials and Methods

### Mice

GF mice (BALB/cYit) were maintained for generations, and specific SPF mice were prepared from GF littermates by transferring into SPF fostered mothers immediately after birth at the Yakult Central Institute. For FACS analysis, GF and SPF mice were purchased from Sankyo Lab. Nod1^−/−^ mice [8] were kindly provided by Dr. Mak (University Health Network) and were bred at the animal facility of Juntendo University. Pregnant BALB/c mice for fetal thymus organ culture (FTOC) were purchased from SLC. Mice were sacrificed by isoflurane anesthesia. All animal experiments were approved by the Animal Experimentation Committee of Juntendo University.

### Reagents and antibodies

Staphylococcal enterotoxin B (SEB) was purchased from Toxin Technology. C12-iE-DAP was purchased from Invivogen. For flow cytometric analysis and TEC sorting, mouse monoclonal antibodies against CD8- FITC (5H10-1), CD4-PE (RM4-5), CD4-APC (RM4-5), I-A/I-E-Pacific Blue (M5/114.152), and RANKL-biotin (IK22-5) were purchased from Biolegend, CD45-APC (SB1), I-A^b^-FITC (AF6–120.1), and I-A^d^-FITC (AMS-32.1) were purchased from BD Bioscience, Aire-Alexa Fluor 488 (5H12), rat IgG2c-Alexa Fluor 488 isotype control (RTK4174), and Streptavidin-PE were purchased from eBioscience, and Ulex Europaeus Agglutinin 1-biotin (UEA1) was purchased from Vector Labs.

### Flow cytometric analysis

Whole thymic cells were prepared by enzymatic digestion with collagenase D and DNase I. Cells were incubated for 30 min at 4°C under protection from light with a combination of appropriate fluorescently labeled mouse-specific antibodies. After washing with 1% FBS/PBS, the cells were analyzed by FACS Calibur and FACS Verse (BD Bioscience). CD45^–^enriched cells were first stained with I-A/I-E-Pacific Blue, CD45-APC and UEA1-biotin, and then stained with Streptavidin-PE. Next, for intracellular Aire staining, the cells were fixed with Foxp3 staining kit (eBioscience) in accordance with the manufacturer’s protocol and stained with Aire-Alexa 488 or control antibody. Data were analyzed with FlowJo (Tomy Digital Biology).

### Purification of thymic epithelial cells (TECs)

The cell suspension of thymic tissue was prepared by enzymatic digestion with collagenase D (Roche) and DNase I (Roche) as described [9]. For TEC isolation, CD45^−^ cells from total cells of thymus tissues were first enriched by depleting CD45^+^ cells using a magnetic cell sorter (Miltenyi Biotec). Consequently, these enriched CD45^−^ cells were stained with APC-conjugated CD45 (SB1) and FITC-conjugated antibody specific for I-A^b^ or I-A^d^, and the stained CD45^−^I-A^+^ cells were isolated to be TECs by FACS Aria (BD Bioscience). To detect the proportion of isolated TECs expressing a medullary TEC (mTEC) marker, CD45^−^I-A^b+^ or CD45^−^I-A^d+^ TECs cells were stained with biotinylated-Ulex Europaeus Agglutinin 1 (UEA1) accompanied with streptavidin-PE. For isolation of TECs from FTOC, the cultured thymic lobes were washed and subjected to enzymatic digestion as described above for adult thymus.

### RNA isolation and quantitative mRNA analysis

Total RNA was isolated with an RNeasy micro-kit and RNeasy Mini kit (QIAGEN) in accordance with the manufacturer’s instructions. Complementary DNA was reverse-transcribed with RivaTra (Toyobo). Real-time PCR for quantification was performed with SYBR green (ABI) according to with the manufacturer's protocol, with 40 cycle of amplification. Data were estimated from the relative quantification of target genes and mean ± SD was estimated. The Hprt gene was used for normalization. For PCR amplification, cDNA obtained from sorted TECs was amplified with HotStarTaq (QIAGEN). The primers were as follows: mouse Aire, 5′-ACCCAACAAGTTCGAAGACCC-3′ and 5′-GACAGCCGTCACAACAGATGA-3′; Spt1, 5′-CTGCTGGTGAAAATACTGGCTCTG-3′. and 5′-GCCTCATTAGCAGTGTTGGTATCATC-3′; Ctsl, 5′-CTGTTGCTATGGACGCAAGC-3′. and 5′-CAGAACCCCATGGTCGAGG-3′; RANKL, 5′-AGCCGAGACTACGGCAAGTA-3′. and 5′-GCGCTCGAAAGTACAGGAAC-3′; Crp, 5′-TACTCTGGTGCCTTCTGATCATGA-3′ and 5′-GGCTTCTTTGACTCTGCTTCCA-3′; S100a8, 5′-TCCAATATACAAGGAAATCACCA-3′ and 5′-ATTTATATTCTGCACAAACTGAGG-3′; Expi, 5′-CAGCCACAGTCTTTGTTCTGG-3′ and 5′-CAGAGCACGATCCATCTCC-3′; Nod1, 5′-GCGAGGAGGTGTCTGAGTTC-3′ and 5′-ATAGGTCTCCTCCAGCAGCA-3′; Nod2, 5′-CCCTGGCTGAAGTTGTAGC-3′ and 5′-GAGTTCCTCTAGTGACTTG-3′; mouse Hprt, 5′-GCAGTACAGCCCCAAAATGG-3′ and 5′-AACAAAGTCTGGCCTGTATCCAA-3′.

### Fetal thymus organ culture (FTOC)

FTOC was performed as previously described [10]. Briefly, BALB/c fetal thymic lobes were removed from embryos (E17) and each right and left lobe removed from one embryo was divided into a separate group to culture on Nucleopore filter (Whatman) floating on RPMI 1640 medium (Sigma) supplemented with 10% fetal calf serum (Sigma), 500 U/ml penicillin, 500 µg/ml streptomycin, 10 mM Hepes buffer, and 1 mM 2-mercaptoethanol in the presence or absence of C12-iE-DAP or SEB. For the 2-deoxyguanosine (dGuo)-treated FTOC experiment, fetal thymic lobes (E16) were cultured in dGuo (1 mg/ml) for 4 or 6 days, and after washing, they were re-cultured with reagents for another 2 days.

### Statistical analysis

Statistical analysis was performed using student’s t test (GraphPad Prism 4). We considered P values of <0.05 as significant.

## Results

### Aire expression in TECs of SPF and GF mice

**Figure 1:**
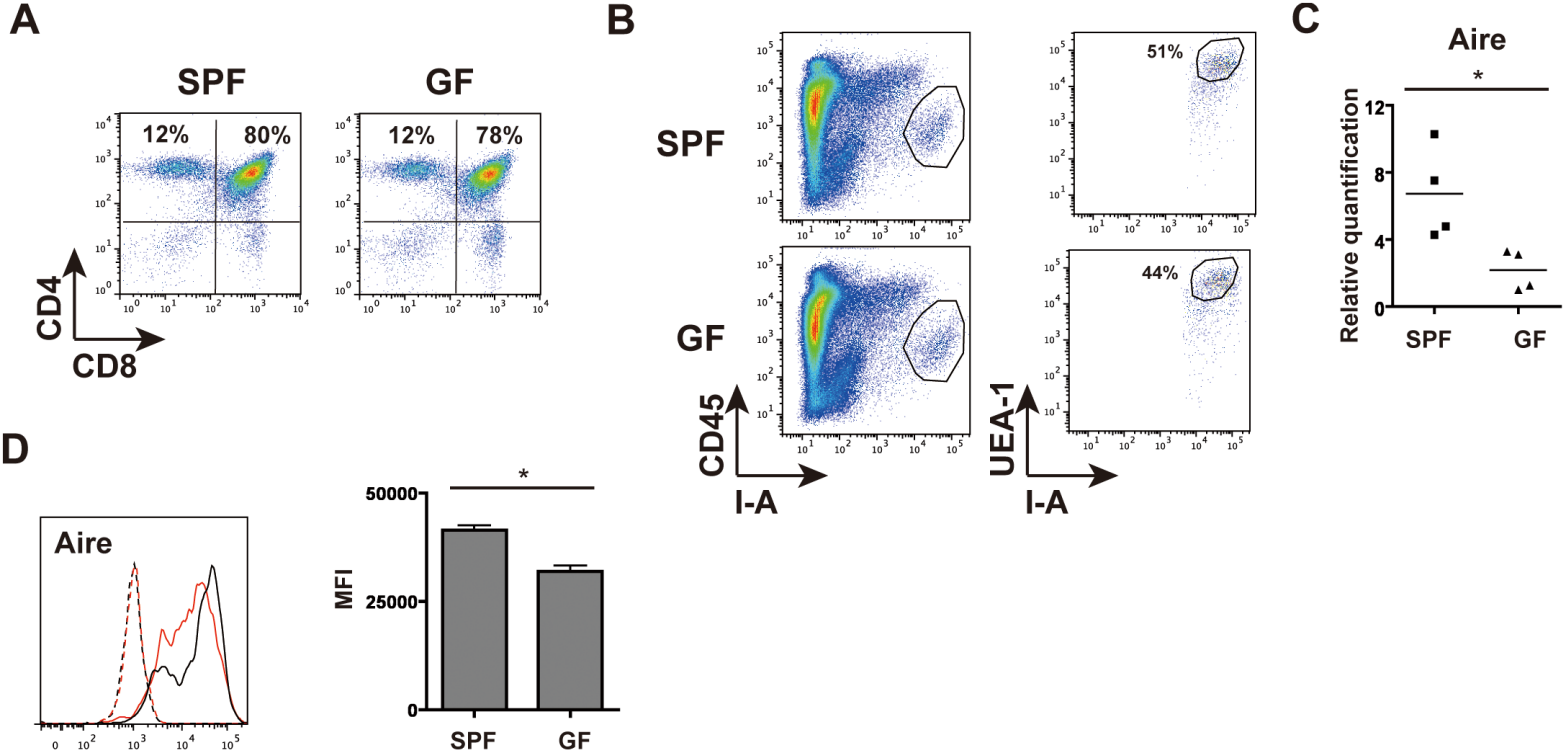
Lower Aire expression in TECs of GF mice. (A) Thymocytes obtained from SPF and GF mice were stained with antibodies against CD4 and CD8. Cell numbers and proportions of CD4/8 subsets were almost equal in the two mice. (B) After roughly enriching CD45^−^ cells by depleting CD45^+^ cells from whole thymic cells using AutoMACS, they were stained with anti-I-A^d^-FITC, CD45-APC and UEA1-biotin and then Streptavidin-PE. Circulated areas in GF and SPF mice in left panels were isolated by FACS Aria to be CD45^−^I-A^+^ cells, and they were used for real-time PCR analysis. In separate experiments using 4 GF and SPF mice each, circulated areas were further analyzed for the proportions of UEA1^+^I-A^+^ cells in CD45^−^I-A^+^ cells. The average proportions for gated UEA1^+^I-A^+^ cells in CD45^−^I-A^+^ cells from independent experiments are shown in the right panel. (C) CD45^−^I-A^+^ cells isolated as TECs from SPF and GF mice were analyzed for Aire mRNA expression by real-time PCR. Relative quantification of gene expression is shown as mean of values from triplicate samples, and the lowest Aire expression level in GF was arbitrarily set at 1. The relative gene expression level is shown for individual TECs from 4 heads each of GF and SPF mice. *P<0.05. (D) For FACS analysis of Aire experiments, enriched CD45^−^depleted cells were first stained with CD45-APC and UEA1-biotin facilitated with Streptavidin-PE, and anti-I-A/I-E-Pacific Blue (to distinguish the anti-Aire reaction that was conjugated with Alexa Fluor 488). Then, for intracellular Aire staining, the cells were fixed as described in Materials and Methods and stained with Aire-A488 or control antibody. Representative FACS plots of Aire expression of SPF (black line) and GF (red line) mice are shown. Black and red dotted lines indicate isotype controls of SPF and GF mice, respectively. The mean fluorescence intensity (MFI) for Aire from three independent experiments is shown (right). *P<0.05.

Accumulating evidence has indicated that commensal bacteria in the intestine regulate local responses mediated by multiple immune cells such as IL-17-producing T cells and Treg cells [1], [3]. However, most reports focused on lymphoid tissues limited to the local area, not on those distant from the intestine. In this study, we chose the thymus to examine whether commensal bacteria can exert any influence on remote lymphoid tissues, because the thymus contains mainly intrathymically differentiated T cells but not T cells exposed to the bacteria. In order to address the effects of commensal bacteria on the thymus, we compared GF and SPF mice; half of the mice bred in GF isolators were transferred to SPF condition immediately after birth and were fostered with SPF mice. Thymocytes of 6-week-old GF mice and SPF littermates were submitted to flow cytometric analysis. The results showed no significant differences in thymocyte cell number (SPF; 2.1±0.3×10^8^, GF; 2.2±0.2×10^8^) or subsets marked by CD4 and CD8 (SPF; DN: 3.1±0.2%, DP: 77.1±2%, 4SP: 13.8±1%, 8SP: 5.1±0.8%, GF; DN: 3.5±0.5%, DP: 77.8±0.3%, 4SP: 13.7±1.7%, 8SP: 4.9±0.6%; Fig. 1A).

Then, we tried to examine whether commensal bacteria can somehow exert influence on the thymic microenvironment. For that, we isolated CD45^−^I-A^+^ cells as TECs by the process described in Materials and Methods. In fact, the isolated CD45^−^I-A^+^ cells contained mostly equal proportions of cells stained with Ulex Europaeus Agglutinin 1 (UEA1), a marker of mTECs, in both SPF and GF (52±7% and 49±5%, respectively; Fig. 1B). Interestingly, Aire expression in TECs detected by real-time PCR analysis was reduced in GF mice compared to SPF littermates (Fig. 1C). In separate experiments, Aire expression in UEA1^+^I-A^+^ TECs was examined using flow cytometry. Although the expression difference between the two groups was not large (Fig. 1D, left), mean fluorescence intensity (MFI) was significantly reduced in GF mice (Fig. 1D, right), in correlation with the mRNA expression. These results suggested a certain contribution of commensal bacteria to Aire expression in TECs.

### Low Aire expression in Nod1^−/−^ mice

**Figure 2:**
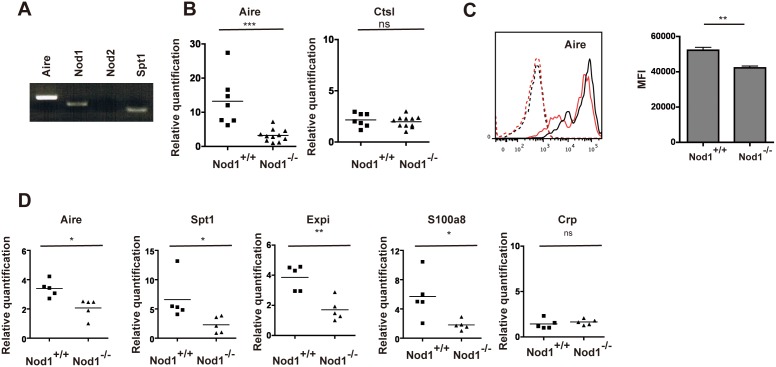
Lower Aire expression in TECs of Nod1^−/−^ mice. (A) Gene expression of TECs isolated from 5-wk-old wild type mice was examined by PCR. (B) From littermates, TECs were isolated individually from 7 heads of Nod1^+/+^ (WT) mice and 11 heads of Nod1^−/−^ mice. mRNA expression was estimated as described in Fig. 1C. The lowest Aire or Ctsl expression level in Nod1^−/−^ mice was arbitrarily set at 1. ***P<0.001. (C) For FACS analysis, the Aire expression on TECs (UEA1^+^I-A^+^CD45^−^) in I-A^+^CD45^−^ cells of Nod1^+/+^ and Nod1^−/−^ mice was measured as described in Fig. 1D legend. Representative FACS plots of Aire expression of Nod1^+/+^ WT (black line) and Nod1^−/−^ (red line) mice are shown. Black and red dotted lines indicate isotype controls of Nod1^+/+^ and Nod1^−/−^ mice, respectively. MFI for Aire from three independent experiments is shown (right). **P<0.01. (D) Expression of Aire-dependent (Spt1, Expi and S100a8) and -independent (Crp) genes in TECs from Nod1^+/+^ (n = 5) and Nod1^−/−^ (n = 5) mice were analyzed by real-time PCR as described in Fig. 1C. *P<0.05, **P<0.01.

Then, based on the above results showing the decreased Aire expression in TECs of mice free of commensal bacteria, we further examined Aire expression in TECs using mutant mice lacking Nod1 in order to confirm whether commensal bacterial products are somehow involved in Aire expression in the thymus. Nod1 is a member of the NLR protein family and an intracellular pattern recognition receptor specific for bacterial components such as the bacterial cell wall, PGN, mainly produced by Gram-negative bacteria [11]. Thus, Nod1-deficient (Nod1^−/−^) mice are predicted to have limited recognition of bacterial components and to reveal a phenotype close to GF mice. Before comparison of Aire expression in TECs, we first checked whether Nod1 was expressed in TECs, and showed that Nod1 but not Nod2 was expressed in isolated TECs (Fig. 2A).

Then, we prepared 5-week-old Nod1^−/−^ and Nod1^+/+^ wild-type (WT) mice bred under SPF condition. As seen in the comparison of GF and SPF mice, thymocytes of Nod1^−/−^ and WT mice did not differ in overall cellularity (Nod1^+/+^; 2.3±0.4×10^8^, Nod1^−/−^; 2.6±0.4×10^8^), CD4/8 subsets (Nod1^+/+^; DN: 4.2±0.8%, DP: 81.1±1.3%, 4SP: 10.6±1.4%, 8SP: 4.0±0.4%, Nod1^−/−^; DN: 4.7±0.7%, DP: 80.3±1.2%, 4SP: 12.0±0.7%, 8SP: 3.5±0.3%). However, as expected, isolated TECs from Nod1^−/−^ mice and WT mice revealed different Aire expressions in terms of mRNA level between them: lower Aire in Nod1^−/−^ mice than in Nod1^+/+^ WT mice but not different in Cathepsin L (Ctsl), a marker of cortex TEC expression (Fig. 2B). Flow cytometric analysis of Aire expression was also carried out. The histogram of Aire protein expression in UEA1^+^I-A^+^ cells showed a small difference between WT and Nod1^−/−^ mice (Fig. 2C, left), but MFI of the FACS profile was significantly decreased in Nod1^−/−^ (Fig. 2C, right), consistent with the mRNA expression. In addition to Aire, mRNA expression of the Aire-dependent genes such as Spt1, Expi and S100a8 [12], [13] was also down-regulated (Fig. 2D), whereas that of Crp, an Aire-independent gene [14], did not differ (Fig. 2D).

### Nod1 ligands enhance Aire expression in TECs directly

The above in vivo features of Nod1 mice suggested that Aire expression seemed to be partly regulated by components of commensal bacteria that are recognized by Nod1 in TECs after their migration from the intestine into the thymus. In fact, recent reports showed that bacterial products including PGN are circulating in serum [4], [7].

**Figure 3:**
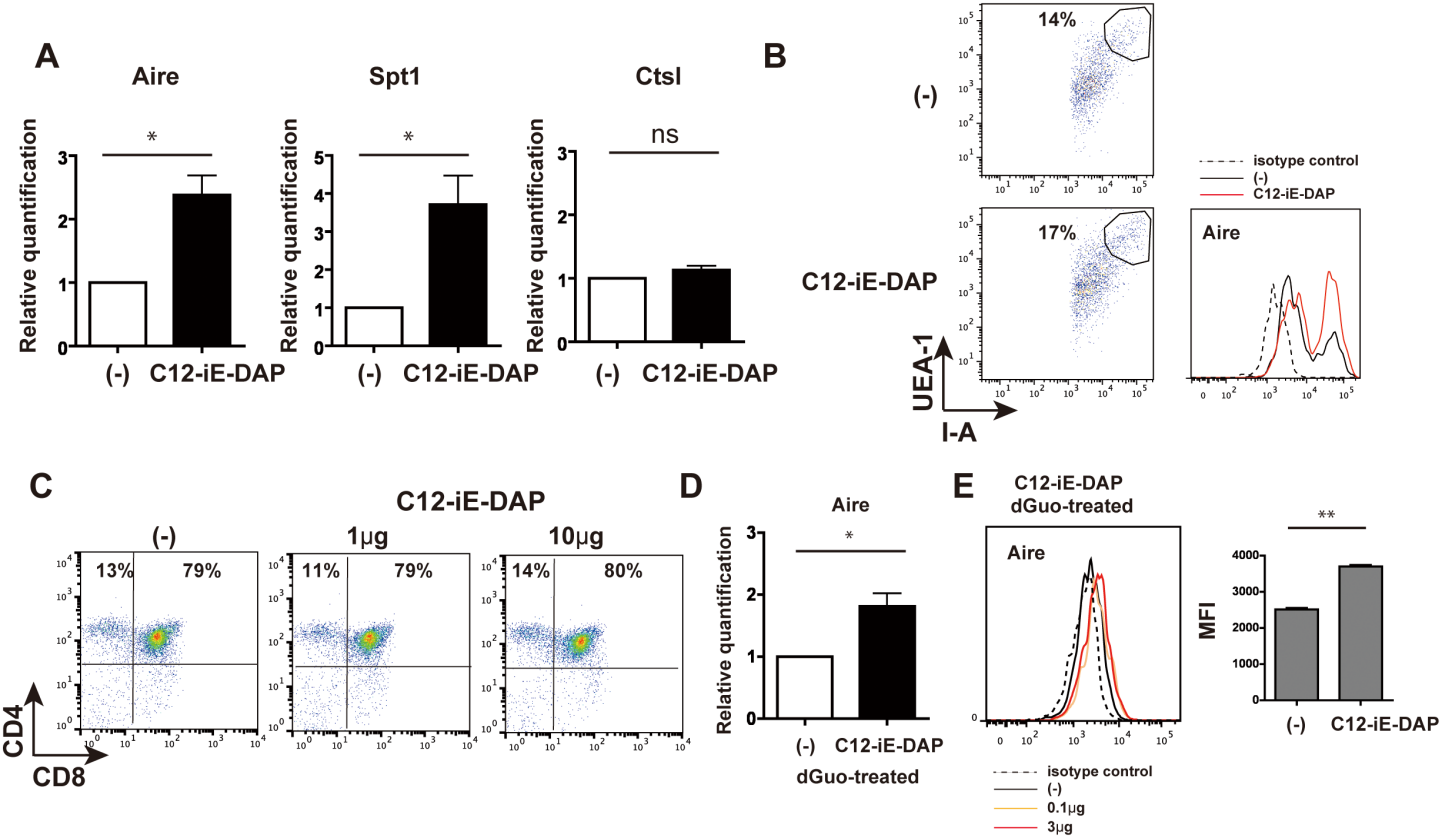
Aire induction by synthetic Nod1 ligands in TECs. (A) E17 BALB/c thymic lobes removed from 3∼5 mothers were separated into right and left lobes and cultured on filter membranes for right and left lobes, and then cultured with and without C12-iE-DAP (10 µg/ml). After 3-day culture and washing, TECs were isolated from thymic lobes by the same procedure as described in adult thymus. Gene expression of the isolated TECs was analyzed by real-time PCR to test Aire expression. In all panels, data were estimated from the relative quantification, and the untreated samples were arbitrarily set at 1. Data bars represent mean ± SD of three independent experiments. *P<0.05. (B) For FACS analysis in the experiment similar to the above (A), Aire expression in cell suspensions obtained from FTOC with and without C12-iE-DAP (10 µg/ml) was manipulated and measured in gated UEA1^+^I-A^+^CD45^−^ cells by the same procedure as described in Fig. 1D legend. Aire expression was clearly increased in TECs receiving C12-iE-DAP (10 µg/ml). For UEA1^+^I-A^+^CD45^−^ cells, a similar intensity of stained plot in the adult TECs was gated (left). (C) Thymocytes from FTOC stimulated with or without C12-iE-DAP were stained with antibodies against CD4 and CD8. Panels show representative results and percentages of DP and 4SP cells in four independent experiments. (D) After removing lymphocytes by 4-day FTOC culture with dGuo, thymic lobes were washed and re-cultured with C12-iE-DAP (3 and 0.1 µg/ml) for 2 more days. Such manipulated thymus was used to isolate TECs by the same process as described in all experiments. Then, Aire expression of TECs was analyzed by real-time PCR. Data were estimated from the relative quantification, and untreated samples were arbitrarily set at 1. Data bar represents mean ± SD of three independent experiments. *P<0.05. (E) FACS analysis was performed in the same experiment using separate thymi. The staining process for Aire expression in gated UEA1^+^I-A^+^CD45^−^ cells was similar to that described in Fig. 1D. Representative FACS plots and MFI for Aire expression from three independent experiments are shown. **P<0.01.

To prove this, we examined whether commensal bacterial components might function as a Nod1 ligand to induce Aire expression in TECs. For this purpose, we adopted an FTOC system, in which 17-day fetal thymic lobes were cultured with a Nod1 ligand, C12-iE-DAP, which is synthesized as part of the PGN of Gram-negative and Gram-positive bacteria. After 48–72 hrs of FTOC in the presence of C12-iE-DAP, the cultured lobes were washed and submitted to TEC isolation in a process similar to that performed in the adult thymus. The isolated TECs were subjected to Aire expression determination at the mRNA and protein levels. The results showed increased Aire expression in TECs by co-culture with C12-iE-DAP (Fig. 3A) at the mRNA level, which was accompanied by increased Spt1 and unchanged Ctsl (Fig. 3A). FACS profiles of UEA1^+^I-A^+^ TECs after FTOC stimulated with or without C12-iE-DAP showed the clearly increased Aire expression in the C12-iE-DAP-treated group (Fig. 3B), meaning that Aire was induced by C12-iE-DAP. However, CD4/CD8 subsets of thymocytes were not changed with or without C12-iE-DAP (Fig. 3C).

The cytokine RANK ligand (RANKL) was reported to be expressed on positively selected thymic T cells and to play a major role in increasing the number of mTECs expressing Aire through RANK on TECs [13], indicating that RANK- mediated signaling can induce Aire expression. Thus, to clarify the direct impact of Nod1 ligands on Aire expression in TECs, the possible contribution of thymic T cells should be avoided. For this, thymic lobes were first cultured in the presence of 2-deoxyguanosine (dGuo), which is known to deplete non-epithelial cells but leave TECs intact, to eliminate intrathymic T cells. After treatment with dGuo for 4–6 days and subsequent washing, thymic lobes without T cells were re-cultured with or without C12-iE-DAP for 2 more days. TECs isolated from dGuo-treated thymic lobes showed significantly higher Aire expression at the mRNA level when C12-iE-DAP was added for a subsequent 2-day culture (Fig. 3D and E), although without C12-iE-DAP in the additional 2-day culture, Aire expression was low overall, presumably because TECs were freed from RANKL-expressing T cells for longer culture (4 or 6+2 days) duration.

These results indicated that the components of commensal bacteria, at least PGN, could induce Aire expression in TECs directly through Nod1-mediated activation but not through thymocyte-mediated signaling.

### SEB induces Aire expression in a RANK/RANKL-dependent fashion

**Figure 4:**
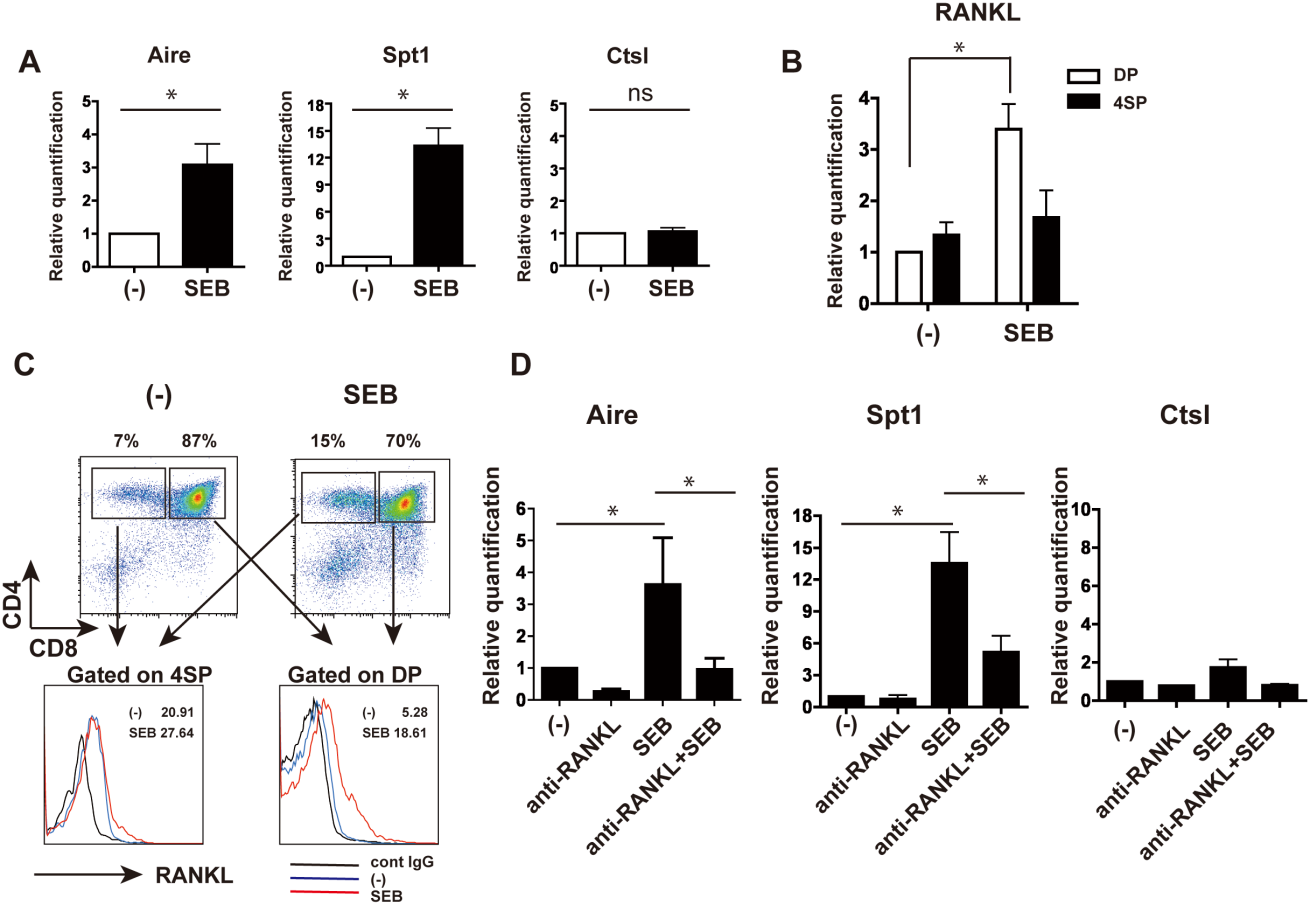
SEB induces Aire expression through RANKL. (A) TECs were isolated from E17 BALB/c thymic lobes subjected to FTOC with or without SEB (10 µg/ml) for 3 days, and their gene expression was analyzed by real-time PCR. (B) RANKL expression in DP and 4SP cells isolated from FTOC was analyzed by real-time PCR. (C) In upper panels, thymocytes obtained from FTOC were stained with antibodies against CD4 and CD8, and analyzed by flow cytometry. The absolute cell numbers in three independent experiments were as follows. Total cell number, untreated: 1.1±0.5×10^7^, SEB: 1.9±0.4×10^6^; DP, untreated: 3.4±1.2×10^5^, SEB: 2.6±1.1×10^4^; 4SP, untreated: 5.0±3.0×10^4^, SEB: 1.1±0.3×10^4^. Percentages of DP and 4SP cells are shown. Lower panels show the flow cytometric profiles of DP and 4SP cells further stained with anti-RANKL or control antibody. MFI is indicated in the panels. (D) TECs isolated from 3-day FTOC with or without SEB in the presence or absence of anti-RANKL antibody were submitted to gene expression analysis by real-time PCR. In all panels of (A), (B) and (D), the untreated samples were arbitrarily set at 1 and data bars represent mean ± SD of at least three independent experiments. *P<0.05.

We also examined the effect of other bacterial products on Aire expression in TECs. As mentioned above, Aire expression is inducible in TECs by interaction with thymocytes expressing RANKL [13]. Thus, if the bacterial components enhance RANKL expression, they may also contribute to inducing Aire expression in a way independent from Nod1-mediated activation. To address this possibility, we selected SEB, an enterotoxin produced by *Staphylococcus aureus*, belonging to Gram-positive intestinal microbiota. SEB is known to function as a superantigen and can strongly cross-link between T cells and MHC class II-expressing cells such as TECs by binding to both TCRβ chain and MHC class IIα chain [15]. To test the SEB effect on Aire expression in TECs, SEB was added to the FTOC system for 3 days, and TECs were isolated as described above and submitted to real-time PCR analysis. As shown in Fig. 4A, Aire expression in TECs was up-regulated with SEB stimulation. Aire-dependent gene Spt1 was also up-regulated, but no difference in the expression of Ctsl was observed (Fig. 4A). In thymocytes, SEB stimulation decreased the absolute cell number and clearly increased RANKL in the remaining thymocytes, and particularly in DP cells (Fig. 4B and C). In comparison, without SEB, it is possible that a larger proportion of DP cells including positive and negative selected and non-selected cells are able to be activated by binding through SEB between MHC IIα chain and multiple types of β chain of TCR, and consequently many DP cells may express RANKL. In contrast, CD4^+^ cells may receive SEB influence less effectively probably because they are already selected and matured under MHC-restricted repertoire in FTOC with and without SEB.

To determine whether the Aire expression in TECs in the presence of SEB was preferentially dependent on RANKL-mediated RANK signaling, blocking antibody against RANKL was added to FTOC with SEB. The result showed that the enhanced Aire expression by SEB was largely abrogated nearly to the control level by anti-RANKL antibody (Fig. 4D). Spt1 was also down-regulated by adding anti-RANKL antibody with SEB, whereas Ctsl was not changed (Fig. 4D).

These results imply that a certain bacterial product such as SEB can induce increased Aire expression in TECs mainly through enhanced RANKL expression on T cells by widely cross-linking between DP cells and TECs, resulting in the induction of enhanced RANK signaling, leading to Aire expression.

## Discussion

The present study showed that Aire expression in TECs was decreased in GF mice without commensal bacteria and in Nod1-deficient mice lacking a sensor for certain bacteria. These in vivo findings suggest that there was, somehow, a contribution of commensal bacteria to Aire expression in TECs under physiological condition. Indeed, substantial effects of bacterial components on Aire expression in TECs were demonstrated in the organ culture system FTOC, in which Nod1 ligand represented by a synthetic moiety that is part of the structure of Gram-negative and -positive bacterial cell walls could enhance Aire expression in TECs. Since ligand-stimulated Nod1 can activate the NF-κB transcription factor down-stream [16] and lead to multiple gene expression including Aire [17], [18], the enhancement of Aire expression in TECs seems to be mediated by the direct effect of Nod1 ligands through Nod1. FTOC experiments with a product of *Staphylococcus aureus*, SEB, showed the induction of Aire expression in TECs in a distinct way. In FTOC with SEB, RANKL expression in thymocytes was increased (Fig. 4), and consequently RANK signaling in TECs might be more activated by stronger RANKL/RANK interaction. Because RANK signaling is important for the development of mTECs expressing Aire [13], [18], our results suggested that Aire expression is controlled by commensal bacterial components in at least two ways, RANK-dependent (probably indirect) and Nod1-dependent (direct) processes.

The previous study revealed that Nod1 is required for the development of gut lymphoid tissue by commensal bacteria [19], but our study is the first to report that commensal bacterial products cause some effect on epithelial cells of the thymus distant from the intestine, besides on the limited local area. The reagents we used were SEB produced by *Staphylococcus aureus*, belonging to Gram-positive intestinal microbiota, and C12-iE-DAP synthesized as part of the PGN of Gram-negative bacteria and probably Gram-positive ones. Both are products or components of bacteria themselves but not their metabolic or digestive products from food. Moreover, they can induce signaling preferentially in epithelial cells through highly expressed RANK on thymocytes or through Nod1 recognizing PGN probably migrated into the thymic tissues. Recent studies demonstrating PGN in circulating serum may support the present study [7]; commensal bacterial products have some effect on the thymus, at least on epithelial cells.

In the mice we analyzed for Aire expression, whole thymocytes and their subsets were not changed in GF and Nod1^−/−^ mice in compared to SPF-conditioned wild-type ones despite the Aire reduction. A similar phenotype of thymocytes was also reported in Aire-deficient mice, although those mice were reported to show autoimmune phenotypes in several organs [20]. In Aire ^+/−^ mice, however, no sign of autoimmune reaction was reported [21], although our examination showed that Aire expression of their TECs was reduced (data not shown). Taken together, it is suggested that, for induction of autoimmune phenotypes, a complete or at least a major deletion of Aire expression may be required, whereas a relatively mild reduction may not be sufficient. Further studies will be needed for coupling Aire expression in TECs to the induction of autoimmune diseases.
